# Advancing Wider Implementation of Multi-regional Clinical Trials in East Asia

**DOI:** 10.1007/s43441-026-00981-5

**Published:** 2026-05-05

**Authors:** John H. Skerritt, Irene Chan, Aloka Chakravarty

**Affiliations:** 1 Faculty of Medicine, Dentistry and Health Sciences, University of Melbourne, Melbourne, VIC 3010 Australia; 2 Eli Lilly and Co, 306 East Merrill St, Indianapolis, IN 46285 USA

**Keywords:** Clinical trial, Regulation, ICH E17, Ethnic differences, Patient access

## Abstract

The ICH E17 guideline is intended to promote the effective and efficient use of multi-regional clinical trials in simultaneous global medicines development, contributing to faster patient access. However, its uptake has been slower than hoped, and some regulators often require local (national) clinical trial data for regulatory submissions. This review examines the extent of ethnic (country of origin) influences on pharmacokinetic (PK) or pharmacodynamic (PD) profile of drugs, with a focus on East Asian countries. We conclude that clinically significant ethnic differences are likely to be less common for many newer therapeutics than older drugs, especially those older small-molecule drugs with low therapeutic indices. Major source of ethnic differences in drug PK or PD are due to differences in the allelic frequencies of particular genes for metabolizing enzymes and other drug targets or receptors, respectively. The default approach for conducting clinical trials for products intended for global markets should be to assess safety and efficacy through multi-regional clinical trials and requirements for country-specific enrolment in trials infrequently justifiable only on scientific grounds.

## Introduction

Medicines development is increasingly a global undertaking and a central tool to enable regulatory agencies globally to consider submissions for new drugs within similar timeframes has been the introduction of multi-regional clinical trials (MRCTs) supported by the E17 guideline of the International Council for Harmonisation of Technical Requirements for Pharmaceuticals for Human Use (ICH) [1].

In addition, there is increasing use of Model-Informed Drug Development [2], integrating nonclinical and clinical data, prior information, and knowledge on the drug and disease characteristics to generate evidence. Use of PK/PD modeling with covariates, as outlined in the ICH M15 guideline, can be used to address differences, reducing the need for standalone studies.

The basic assumption underpinning the conduct of MRCTs is that the evident treatment effects will apply to the entire target population and that clinical trial subpopulations can be defined in regions rather than individual countries. Notwithstanding the adoption of ICH E17 by most major regulatory agencies since 2017, certain regulators often require local (national) clinical trial data in many submissions. The main focus of this review is on East Asian countries, the extent of their implementation of ICH E17, and the potential for greater adoption of ICH E17 principles such as regional pooling.

This article assesses the extent of ethnic differences in PK, PD or efficacy and safety of drugs and whether, particularly for newer therapeutics, such impacts are common or clinically significant in many cases. We conclude that local (country-specific) patient population study requirements are appropriate only where there is a strong scientific rationale based on known or anticipated regional or ethnic differences identified through early clinical studies or from an understanding of the response to similar therapeutics.

## Variable Implementation of ICH E17 in East Asian Countries

There has been a spectrum of regulatory expectations on regional patient representation across East Asia to date. In China, for medicines whose safety and efficacy have been verified internationally, a bridging study [3] with local patients is required, although conditional approval is possible if the product treats a life-threatening condition and is superior to existing therapies [4].The China National Medical Products Administration Center for Drug Evaluation (CDE) recently released Guidelines for Benefit-Risk Assessment Based on Multi-Regional Clinical Trial Data in Global Simultaneous Development of New Drugs [5]. These guidelines aim to support companies in integrating MRCT data into benefit-risk assessments, aligning China’s regulatory framework with international standards. Similar provisions apply in Taiwan with guidance published on the conduct of bridging trials with local participants [6]. PMDA’s guidance [7] states that Japan can participate in MRCTs without a phase 1 study in Japan for “drugs with high unmet medical needs, such as drugs for rare diseases, diseases that are refractory and serious, or pediatrics” or if the safety of Japanese participants, can (otherwise) “be judged to be clinically acceptable”.

In Japan, guidance released in 2007 [8], some years before the adoption of ICH E17 recommends that MRCTs include a sufficient number of Japanese subjects to ensure either a likelihood of consistency in efficacy and safety between the overall population and the Japanese subpopulation, or a likelihood of similar trends across multiple regions. Written regulatory requirements, such as in South Korea [9], do not typically require specific numbers of local trial participants; however, these are often communicated by the regulator to the trial sponsors. While it is the prerogative of national regulators, these local requirements can lead to delays in patient access to important new therapeutics. Conduct of bridging studies that investigate whether ethnic factors influence PK and comparative dose-response can eliminate the need to conduct full local phase III trials but may still delay regulatory submission and subsequent patient access [10].

The uptake of ICH E17 has been slower and less consistent between countries than expected. Apart from regulatory inflexibility, a recent review [11] attributes this to slow adoption of recommended pooling strategies in clinical trials, ambiguity in the definition of a region in MRCT, the use of decentralized clinical trials and data sources such as Real World Data and the impact of the COVID pandemic. Governments such as Japan have supported collaborative efforts and have undertaken regulatory reforms to encourage MRCTs [12, 13]. While several factors contribute, inconsistent or incomplete implementation of ICH E17 principles mean that “drug lag”, where new drugs are submitted for regulatory review sometime later than in the US or EU (or perhaps not at all) is still significant in several East Asian economies [14–17] and, notwithstanding increased participation rates in MRCTs in Japan and Korea, will likely continue into the future under current policies and practices [18, 19].

Some local surveys of company and clinical research organization users of ICH E17 have also reported that implementation could be increased if the guidelines were more readable and specific examples of MRCT design options including pooling strategies were available in the guidelines [19]. Reasons for slow or incomplete adoption of MRCT may include regulatory conservatism (continuation of policies and practices by regulatory agencies in place prior to formal ICH E17 adoption, or prior to changes in the types of drugs under regulatory review). A requirement for local trials could also formally or informally be a means of encouraging more clinical trials locally. Several countries have launched incentives to encourage more local clinical trials, but these do not usually involve a regulatory requirement to conduct trials locally. For example, there are significant financial incentives for companies to conduct clinical trials in Australia [20]and streamlined regulatory oversight also means that trials can be approved and commenced much earlier than in countries such as the USA [21].

Application of ICH E17 has utility for the full range of medicinal products (drugs and biologics) and to any company wishing to market a new drug or biologic across a reasonably wide range of countries internationally. Lack of adoption of ICH E17 directly impacts patients (and their healthcare providers) because it may result in lack of access to the latest and most appropriate therapeutics.

### Potential Ethnic Differences in PK or PD Response

The ICH E5 and E17 guidelines recognise the importance of “intrinsic factors” affecting dose-related clinical pharmacology – namely PK, PD, dose selection, safety and efficacy [22]. Many of these are affected by genetic polymorphisms, although of the intrinsic factors, differences in gender, age and body mass index (BMI) and comorbidities can influence treatment effects [23–25] but individual differences are more relevant than national average values of these parameters. Even where factors such as national average body mass may differ by 20–30% between countries, this may not be significant enough to justify differences in dosing of oral drugs. The most important intrinsic influence on drug PK/PD may instead be individual pharmacogenetic differences [26, 27]. Suarez-Kurtz [26] concluded that *“inter-individual differences (in genetic polymorphism) account for most of all human variation…this paradigm extends further to the variation in drug response across populations/ ethnicities”.*

“Extrinsic factors” such as differences in epidemiology of particular diseases and in approaches to disease diagnosis, classification and managed care including concomitant medications in different regions can be important. However, several of the extrinsic factors (e.g. socioeconomic status, diet, alcohol/tobacco use, drug compliance) relate more to individual lifestyle factors than ethnicity (i.e. country of origin) [28, 29]. In contrast, in some cases national differences in medical practice, availability and use of comparator and concomitant medications, trial design and availability of testing can more consistently influence possible national differences in trial results [30, 31]. An analysis of 48 late-stage Alzheimer’s disease trials [32] concluded that subtle differences in clinical endpoints of these trials based on neuroimaging studies conducted in Japan and the USA had a significant effect. It is therefore important, to the extent possible, to ensure that trial design and endpoints are consistent across different countries. This includes that when comparators are used, they are the same in different countries and that trial participants in different countries are screened to enable consistent patient inclusion and exclusion criteria.

If there is evidence that ethnic variability in intrinsic or extrinsic factors is likely to affect response (e.g. because of genetic differences in interaction of the drug class with a drug transporter or metabolizing enzyme) in these cases an evaluation of how it could potentially affect dose-response could be conducted as a component of the phase 2 development program, rather than requiring a bridging study. This approach has been recommended in ICH’s Questions and Answers relating to the ICH E5 guideline [33] and its utility has been confirmed in several case studies of phase 2 trials [34, 35].

### Studies Indicating Differences in PK or PD Between East Asian and Caucasian Populations are Typically of Older Small Molecule Drugs

A significant number of studies, particularly those published in the 1990s and 2000s [36–38] reported differences in metabolism of small molecule drugs between East Asian and Caucasian populations (as well as modest differences in plasma protein binding in some cases [39]). This can lead to increases in drug plasma levels in East Asians compared with Caucasians receiving the same dose [40, 41]. Some examples of small molecule drugs affected include propranolol, morphine, nifedipine, triazolam, diazepam and omeprazole, benzodiazepines, certain anticonvulsants, beta blockers and warfarin and some cytotoxic chemotherapy agents. Recommended doses in East Asia for antipsychotics, lithium and antidepressants are also generally lower than for non-Asians [40].

In an analysis of drugs approved by the US FDA between 2014 and 2019, only 10% showed differences in exposure or response based on known race, ethnicity or pharmacogenetic factors [42], compared with 21% of new drugs approved between 2008 and 2013. The affected drugs were mainly oncology and antiviral small molecule products. Possible requirements for dose adjustments could be related to genetic differences in drug metabolizing enzymes including *CYP2D6*, *CYP29*, *CYP2C19*, *NAT 2* (N-acetyltransferase 2) and *UGTIAI* (UDP – glucuronyl transferase). Drugs that are not metabolized extensively and/or did not show high plasma protein binding were unlikely to demonstrate ethnic differences. Even if individual genetic differences may lead, for example, to a 20–40% difference in serum levels of a particular medicine, for most medicines such differences would rarely be clinically relevant requiring adjustment of the dose. The exception would be for those medicines that have a low therapeutic index. However, in recent years fewer medicines with low therapeutic indices have reached late-stage clinical development [43, 44].

There is also little evidence for ethnic differences in response to biologics, including monoclonal antibody and other therapeutics. Several studies [45–47] suggest that any differences in antibody pharmacokinetics between Japanese and Caucasians are attributable to individual differences in body weight and/or antigen levels. Population PK/PD modeling and analysis also showed no difference in PK and PD of the lipid-reducing monoclonal antibody, evolocumab, between Caucasian and Asian populations [48]. No difference between Chinese and Caucasian or other populations was found in the PK, efficacy or safety of ropeginterferon alfa-2b [49]. The lack of ethnic difference is likely due to these biologics not being metabolized through small-molecule drug pathways such as CYP enzymes and biologics also commonly display “flat” dose-response curves [50, 51]. Whereas over 40% of small molecule drugs approved between 2012 and 2020 had different dosing regimens between Korea and Japan, only just over 10% of monoclonal antibodies did [52].

With biologics accounting for a larger proportion of new drugs in the last decade, it therefore follows that overall, the number of new drugs that show genetic variation in PK or PD should be lower [53]. If the drug is not first-in-class, experience with other drugs in the class can also be instructive [54, 55]. If the sponsor has a good understanding of the metabolic pathways for the drug and the pathways appear unlikely to be affected by genetic variation, this information can be utilized as scientific justification supporting a lack of ethnic difference in drug response, and thus in support of regional pooling under ICH E17 principles.

### Few Studies have Shown Clinically Significant Differences in PK and PD or Safety Between Different East Asian Populations

Studies that have explored potential PK- or PD- related gene polymorphisms among various East Asian populations have found few differences between nationalities [56, 57]. The handful of examples where there are major safety or efficacy differences between Asian groups are well known because of their rarity. Carbamazepine and risk of Stevens-Johnson syndrome and toxic epidermal necrolysis [58] is strongly associated with the HLA-B*1502 gene in Han Chinese. HLA-B*1502 occurs in 10–15% of individuals from southern China, Taiwan and some other Asian economies but is uncommon in Japan and Korea (< 1%) and Caucasians (0–0.1%) [59]. Another example is abacavir-induced hypersensitivity syndrome linked to HLA- B*5701, where there is also some variation between different Asian populations, but overall, a higher frequency in Caucasians. A genetic test is recommended prior to treatment [60].

There are few comparative studies that demonstrate PK differences between East Asian populations. Significant differences in PK and safety among East Asians were not found for four compounds with different mechanisms of action and PK profiles (bazedoxifene, crizotinib, tolterodine and varenicline) [61]. Similarly, there were no significant efficacy differences observed for asenapine and tadalafil observed between Japanese, Korean and Taiwanese populations in clinical trials [62]. Another study also found no PK differences among East Asian populations when clinical trials of three common drugs, moxifloxacin, simvastatin and meloxicam were undertaken under comparable conditions [63]. Similar frequencies of genetic polymorphisms in metabolizing enzymes *CYP2C9*,* CYP2C19*, *CYP2D6* and *CYP3A5* were noted in South-East and East Asian populations [64]. Among Japanese, Chinese and Koreans genetic differences in CYP affecting drug metabolism were negligible or very modest [65, 66]. Equivalent plasma drug concentrations and metabolic profiles were expected for Japanese, Koreans and Chinese populations for drugs metabolized through six major CYP enzymes [67]. Some studies [68] have reported PK differences between Japanese and Korean populations that could be related to differences in mean frequencies in genes for a metabolizing enzyme (CYP2D6*10). However, Singh et al. concluded that individual rather than ethnic differences in drug metabolism by certain cytochrome P450 families are more relevant [69].

### Many Observed Ethnic Differences are Not Related to Ethnicity (Country of Origin) But to Differences in Average Frequencies of Gene Alleles

Differences in allelic forms of drug metabolizing enzymes and transporters (*OATP1B1*, hepatic drug uptake transporter) can affect pharmacokinetics. *CYP2D6*, *CYP2C9* and *CYP2C18* are responsible for initial metabolism of about 40% of small molecule drugs [27]. Genetic polymorphisms which affect pharmacodynamics include *EGFR* (epidermal growth factor receptor and *HLA* (human leukocyte antigen gene complex).

There is a wide variation in estimated prevalence of variants for cytochrome P450 and other genes affecting drug PK or PD of different allelic forms in Caucasian versus East Asian ethnic groups [70–74] (Table 1). However, prevalences often overlap. With a few exceptions, allelic forms that influence drug metabolism (“increased” or “decreased” function) occur at low to moderate frequencies in many populations. Critically, differences in “average frequencies” do not translate to implications for a particular individual. For example, in the situation (see Table 1) where for CYP2C9*3 up to 11% of Caucasians and 5% of East Asians have an allele for fast metabolism, this means that most (up to 89% of Caucasians and 95% of East Asians) are “standard” metabolisers and would not potentially require dose adjustment.

**Table 1:** Reported differences in allelic frequencies of selected genes encoding drug metabolizing enzymes and drug transporters between Caucasian and East Asian populations

Allelic variant	Caucasian frequency	East Asian frequency	Reference
**Drug metabolizing enzymes**			
CYP2C9*2	8–16	0–1	73
CYP2C9*2	8–16	0-0.1	74
CYP2C9*3	4–11	1–5	73
CYP2C9*3	4–10	1–4	74
CYP2C19*2	8–27	6–49	73
CYP2C19*2	8–20	29–50	74
CYP2C19*3	0-6.8	0–21	73
CYP2C19*3	< 1	7–13	74
CYP2C19*17	11–33	0-6.2	73
CYP2C19*17	15–18	< 5	74
SLCO1B1*1A	56	25	74
SLCO1B1*1B	26	63	74
SLCO1B1*5	2	0	74
SLCO1B1*15	16	12	74
VKORC1	42	89	74

Certain alleles do vary more between East Asian and Caucasian populations than others, but not within different East Asian populations. These include certain enzymes active in the initial phase of drug metabolism, including for some older small molecule drugs, such as the *CYP2D6* (catabolizes lipophilic drugs, including some cardiovascular drugs and neuroactive drugs) and *CYP2C* subfamilies (catabolizes neutral to weakly basic or acidic drugs, such as tricyclic antidepressants, benzodiazepines and antidepressants). CYP2D6*10, metabolizing tamoxifen, atomoxetine and tamsulosin occurs at a 42% frequency in Asians but only 3–7% in other populations. Warfarin and small molecule tyrosine kinase inhibitors are metabolized by *CYP2C9* and clopidogrel by *CYP2C19.* Ethnic differences in metabolism of cardiac drugs arising from variation in the frequencies of *CYP2C* and *VKORC1* (vitamin K epoxide reductase complex subunit 1) for warfarin, *CYP2C19* for clopidogrel and statin cellular transport (*SLC1B1*, solute carrier organic anion transporter 1B1) has been reported [74]. Knowledge of the metabolism of other drugs in the same family and the use of PK/PD modeling during the drug development process [75] could help determine whether allelic variation in CYP genes is likely to have a significant impact.

## Application of More Personalized Approaches to Drug Dosage, Where Practical

Several reviews have highlighted a lack of clinical significance in plasma levels even when genetic or ethnic differences in pharmacokinetics are observed or predicted for various drugs [41, 66, 76]. In cases where dose adjustment is important, a personalized medicine approach may often be preferable to dosing based on ethnicity. For those products that are available in two or more approved strengths, physicians typically start with prescribing the lower approved dose and only increase dose if the therapeutic response is inadequate, and the higher dose is well tolerated. Alternatively, a second drug is often added to the regimen. These approaches are widely used with small-molecule drugs for hypertension, lipids, diabetes, respiratory and central nervous system conditions. For narrow therapeutic index drugs, therapeutic drug monitoring [77] may be more relevant than setting a dose based on the patient’s ethnicity.

Greater use of pharmacogenetic testing may be warranted for drugs whose PK, PD or potential for serious adverse effects are known to be highly impacted by particular alleles. Barriers to its wider adoption, however, include high costs of testing and challenges in integrating the technology into clinical workflows [78–80]. Where warranted, pharmacogenetic approaches can provide a more nuanced dosing recommendation for clinicians. The main regulatory ramifications of implementing more widespread pharmacogenetic testing are to validate biomarkers that can be useful in drug development and to include information and recommendations on the relationship between specific gene alleles and the recommended dose in the product “label” or product information document.

The EMA has developed guidelines to support a rigorous framework for pharmacogenomic applications in medicines development, regulatory decision-making, and clinical practice; an updated version is currently under consultation [81]. There are already many cases where the US FDA has required pharmacogenomic information to be included in the product label [82]. A website has also been developed to indicate regulatory agency-approved drug labels containing pharmacogenetic testing information for US FDA, EMA, Health Canada, Swissmedic and PMDA Japan [83]. A recent study, assessing genomic profiles of large numbers of UK and US residents, also demonstrated that different ethnicity groups show differences in the frequency of pharmacogenomic variants that are directly relevant to optimal drug dosing [84]. However, if not included in the drug label it is difficult to influence evidence-based dosing, although national guidelines for this exist in a number of countries [85].

## Other Potentially Contributing Factors

Studies demonstrating ethnic differences in drug PK/PD, efficacy or safety are more likely to be published than “negative” studies. Therefore few of these “negative” studies have been published. Apart from the studies (discussed above) that have demonstrated no ethnic differences in PK and PD for a range of biologics [45–49], studies have been published showing a lack of ethnic differences in the PK of moxifloxacin [86] or PK and PD of ximelagatran and valsartan respectively [87, 88]. An analysis of the PK and recommended doses of 20 drugs from several therapeutic classes in subjects from China, Japan and Korea did not reveal any distinct differences in exposure to these drugs between the populations of these three East Asian countries, reinforcing the ability to use clinical data from these countries mutually [89]. Finally, a study of 620 new molecular entities approved by the US FDA between 2008 and 2023 revealed that only 6.5% reported ethnic differences in PK and even fewer showed ethnic differences in efficacy (0.6%) or safety (1.6%) in the FDA drug labelling [90].

Statistical anomalies may also erroneously lead investigators to conclude that there is a difference between regions or ethnicities in MRCTs when there is not [91]. An examination of consistency of treatment response for the anti-diabetic liraglutide in clinical trials conducted under ICH E17 across several regions [92] showed that while major adverse cardiovascular events differed between regions when hazard ratios were considered, use of Cox proportional regression analysis to assess heterogeneity supported a conclusion that overall there was consistency between regions.

Long et al. [93] described how aspects of clinical trial design or execution could also lead to misleading conclusions about different treatment effects between regions, such as when interim efficacy analyses are conducted but enrolment may be delayed in particular regions; challenges with adaptive clinical trial designs; single arm studies and small sample sizes for rare disease clinical studies. Yusuf and Wittes [94] also reviewed several cases of claimed geographic variation following conduct of randomized controlled clinical trials and concluded that apparent differences in treatment effects may often “simply reflect expected variability arising from the small sample in each country”. They emphasised the importance of performing statistical tests for homogeneity, establishing whether there is biological plausibility for any apparent differences observed and recognising that in some clinical trials (e.g. for infectious disease therapeutics and vaccines), there can be significant differences in event rates in the placebo group from different regions that confounds analysis.

Lower doses are sometimes recommended more frequently for Asian than Caucasian populations on drug labels, but the trends are not consistent and cannot be explained by differences in pharmacokinetics alone [95]. A review of the (often lower) approved doses of 62 mainly antihypertensive and lipid-lowering drugs in FDA/EU versus Japan concluded that the Japanese regulatory review process may place a greater focus on avoiding adverse events, while US review placed greater emphasis on efficacy [96]. An examination of dosages of drugs approved in both Japan and the US between 2003 and 2008 found that doses for anticancer, antiviral and enzyme drugs were similar, but lower doses of many CNS drugs and drugs affecting the endocrine system were approved in Japan compared with the US [97]. The authors considered it to be due to “more careful dose finding processes in Japan for some drug classes.” There is typically greater safety information provided in many drug labels in Korea and Japan for direct acting oral anticoagulants compared with the US and Europe [98] and others concluded that Asian regulators often had a greater emphasis on safety, with limited concordance in label safety information between regulators [99]. Indications for anti-cancer drugs were also “narrower” in Japan than the USA [100]. Thus, in many cases the differences in the approved labelled dose may be due to differences in emphases between regulatory agencies rather than by physiological or genetic factors.

## Pooling Strategies in MRCTs

Pooling strategies in MRCTs involve the aggregation of data from various trial sites to improve statistical power and offer clearer insights into the research question. Some included clinical trial regions may be pooled during the design of MRCTs, when subregions have similar intrinsic and/or extrinsic factors relevant to the disease area and/or drug being studied in the trials. An example could be East Asians living in both the region and the rest of the world, or patients across regions containing a common genetic marker for drug metabolism. The strategy for pooling data across regions and the principles for pooling subpopulations should be specified at the planning stage and described in the protocol. The impact of pooling is to increase sample size for evaluation of regional consistency, geographical regions, countries or regulatory regions can be pooled. Pooling approaches are introduced to enable careful consideration of major intrinsic/extrinsic factors during trial design, prioritise any key factors relevant to the drug so that data can be collected to address consideration of these factors and whether they may have an impact on the potential treatment effect of the drug. Use of pooled subpopulations (by pooling across the global patient population of patients with similar attributes) can also enable sample size allocation beyond the regional distributions that are typically used. If doing so, early scientific discussion and agreement with regulatory agencies is important.

Methodologies for pooling have been reviewed elsewhere. A stepwise approach, involving first identifying the potentially relevant intrinsic and extrinsic factors; second, examining the geographical regions and subregions proposed for pooled regions; and third to form the subgroup. The study design may still need modification at this stage [101]. A review of the ICH E17 recommended approaches for sample size allocation in a Chinese population context noted that each of the approaches had potential shortcomings, such as subjectivity (fixed and proportional approaches), local significance (large sample size), preservation of effect (challenge in determining the proportion required for meaningful treatment effects) and equal allocation (ignoring differences between regions) [102].

The challenge in pooling data is in ensuring consistency and comparability across datasets. Study designs across sites need to be aligned, with consistency in inclusion criteria, treatment protocols, and outcome measures, and with uniform data collection approaches across sites [103–105]. The consideration of principles in the ICH E17 guideline in MRCTs used for earlier drug approval in Japan have been considered. Asano et al. [106] concluded that “regional allocation should have a scientific basis (rather than arbitrary targets), should support the evaluation of consistency, and should provide the information needed to support regulatory decisions”.

## Conclusion

Full uptake of ICH E17 has been slower than hoped by both regulatory agencies and industry. The ICH E17 guidance only provides *“basic principles”* and *“general recommendations in the planning and design of MRCTs*,*”* which has led to differences in interpretation and application. Greater consensus around the interpretation and implementation of ICH E17 may be required to enable efficient clinical trial design, such as in defining a region for pooling, patient pooling strategies and sample size allocations and the utilization of newer clinical trial designs, e.g. adaptive designs. Inconsistencies within and between regions on disease diagnosis, patient inclusion / exclusion criteria or trial endpoints can also lead to differences in local study design and hence inconsistent implementation of ICH E17. Asano et al. [106] emphasized in reviewing challenges in the implementation of the ICH E17 guideline in Japan that it is important in MRCTs for more consistent use of guidelines for disease definitions and primary end point, training/validation on the primary end point and subject selection, sample size allocation, and standardized efficacy and safety information and greater standardization of efficacy and safety measures. Use of newer, more advanced statistical analysis approaches can assist with establishing plausibility for different populations and assessment of internal and external consistency between regions.

We conclude that while some drugs are affected by ethnic differences in PK, PD and rare adverse events, overall, clinically significant ethnic differences are comparatively limited and are likely to be less common for many newer therapeutics than older drugs. A major source of ethnic differences in drug PK and PD was differences in allelic frequencies of genes for metabolizing enzymes and other drug targets. Others have also proposed that in the cases where a range of strengths of a particular drug has been approved in a given market, individualization of dose based on genetics [106–108] rather that applying blanket doses for a particular ethnic group could be a preferred approach in these cases if it can be implemented under the relevant clinical care paradigm.

We conclude the default position for conducting clinical trials for products intended for global markets should be to assess safety and efficacy through MRCTs, and that requirements for country-specific enrolment in trials are rarely justifiable on scientific grounds. Some East Asian regulators and collaborating partners, such as China [109] and Japan have provided guidance demonstrating greater flexibility. In Japan, the PMDA [7, 10] recommends the participation of Japanese patients in MRCTs from the early stages of development but takes a flexible approach to the number of Japanese participants in exploratory MRCTs and has shown flexibility around a requirement for Japanese patients in MRCTs for rare diseases and life-threatening conditions.

Other flexibilities will also be important in facilitating MRCTs in East Asia [110]. In the development of pooling strategies, rather than utilizing fixed proportion or number of participants from different countries, a strategy based on disease incidence and prevalence could be chosen, which could be informed by real world data (RWD) on disease natural history. The choice of pooling strategies depends on knowledge about the relevance of intrinsic and/or extrinsic factors known to potentially affect the treatment effect. Early establishment of PK/PD profile of the new drug including through use of modeling approaches [88, 104] can provide insight into whether there are clinically meaningful differences in intrinsic/extrinsic factors and can reduce the expectation by some national regulatory authorities for inclusion of local patients. If clinically meaningful differences potentially do exist, then bridging studies can be conducted at an early stage of the clinical trial program rather than late in the program as doing so would delay subsequent regulatory submissions in those countries.

More opportunities for early discussions with relevant regulators will improve the ability to align on clinical trial designs, along with an agreement on the acceptability of the size of apparent differences in treatment effects, noting that in many cases, because of low sample sizes in subpopulations, observed differences may not be either statistically or clinically significant. Trial designs should be able to incorporate newer approaches, including decentralized and adaptive trials, use of data sources beyond RCT and enable consistent use of patient reported outcomes across diverse regions. Tohkin et al. [111] also emphasized that sharing scientific data on ethnic factors in Asian populations will result in fuller implementation of the ICH E17 guidelines, such as those on pooling strategies, and that further collaborative training activities on ICH E17 interpretation and implementation are important. RWD obtained in different East Asian countries, and more broadly, on both the natural history and epidemiology of the disease of condition being treated and on the use of the new drug in particular populations (e.g. as part of compassionate access programs) can also potentially inform whether ethnic factors are likely to be relevant. Application of ICH E17 has utility for the full range of medicinal products (drugs and biologics) and for any company wishing to market a new drug or biologic across a reasonably wide range of countries internationally. However, commercial as well as technical considerations will also play a significant role where the cost of additional trials or data gathering may make it less feasible to eventually seek regulatory approval in certain markets. This is particularly relevant to small and medium sized companies, who over the last decade have brought an increasing proportion of drugs and biologicals to the regulatory submission stage [112]. While regulator-specific interpretations of how MRCTs can be implemented should be avoided, provision of more specific globally consistent advice on ICH E17 implementation could potentially be achieved through development of an ICH question and answer document, developed through collaboration between regulators, industry and academia. Such a document could also support greater regulatory convergence, including reliance and worksharing among East Asian regulators, enabling earlier patient access to innovative medicines [113]. Based on this literature review and evolution of ICH E17 implementation, we believe that country-specific requirements should be the exception, and the default approach for conducting clinical trials for products intended for global markets should be to assess safety and efficacy through multi-regional clinical trials.

## Data Availability

No datasets were generated or analysed during the current study.
